# Complete genome sequencing of three human clinical isolates of *Staphylococcus caprae* reveals virulence factors similar to those of *S. epidermidis* and *S. capitis*

**DOI:** 10.1186/s12864-018-5185-9

**Published:** 2018-11-08

**Authors:** Shinya Watanabe, Yoshifumi Aiba, Xin-Ee Tan, Feng-Yu Li, Tanit Boonsiri, Kanate Thitiananpakorn, Bintao Cui, Yusuke Sato’o, Kotaro Kiga, Teppei Sasahara, Longzhu Cui

**Affiliations:** Division of Bacteriology, Department of Infection and Immunity, Faculty of Medicine, Jichi Medical University, 3311-1, Yakushiji, Shimotsuke-shi, Tochigi, 329-0498 Japan

**Keywords:** *Staphylococcus caprae*, *Staphylococcus epidermidis*, *Staphylococcus capitis*, Complete whole genome sequence, Biofilm formation, Nosocomial infection, Adhesin, SCC*mec*

## Abstract

**Background:**

*Staphylococcus caprae* is an animal-associated bacterium regarded as part of goats’ microflora. Recently, *S. caprae* has been reported to cause human nosocomial infections such as bacteremia and bone and joint infections. However, the mechanisms responsible for the development of nosocomial infections remain largely unknown. Moreover, the complete genome sequence of *S. caprae* has not been determined.

**Results:**

We determined the complete genome sequences of three methicillin-resistant *S. caprae* strains isolated from humans and compared these sequences with the genomes of *S. epidermidis* and *S. capitis*, both of which are closely related to *S. caprae* and are inhabitants of human skin capable of causing opportunistic infections. The genomes showed that *S. caprae* JMUB145, JMUB590, and JMUB898 strains contained circular chromosomes of 2,618,380, 2,629,173, and 2,598,513 bp, respectively. JMUB145 carried type V SCC*mec*, while JMUB590 and JMUB898 had type IVa SCC*mec*. A genome-wide phylogenetic SNP tree constructed using 83 complete genome sequences of 24 *Staphylococcus* species and 2 *S. caprae* draft genome sequences confirmed that *S. caprae* is most closely related to *S. epidermidis* and *S. capitis*. Comparative complete genome analysis of eight *S. epidermidis*, three *S. capitis* and three *S. caprae* strains revealed that they shared similar virulence factors represented by biofilm formation genes. These factors include wall teichoic acid synthesis genes, poly-gamma-DL-glutamic acid capsule synthesis genes, and other genes encoding nonproteinaceous adhesins. The 17 proteinases/adhesins and extracellular proteins known to be associated with biofilm formation in *S. epidermidis* were also conserved in these three species, and their biofilm formation could be detected in vitro. Moreover, two virulence-associated gene clusters, the type VII secretion system and capsular polysaccharide biosynthesis gene clusters, identified in *S. aureus* were present in *S. caprae* but not in *S. epidermidis* and *S. capitis* genomes.

**Conclusion:**

The complete genome sequences of three methicillin-resistant *S. caprae* isolates from humans were determined for the first time. Comparative genome analysis revealed that *S. caprae* is closely related to *S. epidermidis* and *S. capitis* at the species level, especially in the ability to form biofilms, which may lead to increased virulence during the development of *S. caprae* infections.

**Electronic supplementary material:**

The online version of this article (10.1186/s12864-018-5185-9) contains supplementary material, which is available to authorized users.

## Background

Staphylococci are major components of the normal microflora in human and animals [1, 2]. Consistent with traditional culture-based analysis, recent microbiome analysis showed that the skin of healthy humans is predominantly colonized by *Propionibacterium*, *Corynebacterium*, and *Staphylococcus* [3–5]. Most of these skin inhabitants, including coagulase-negative staphylococci (CoNS), are considered harmless to healthy humans. In fact, these organisms protect against invasion by other more pathogenic or harmful organisms [6]. However, recent changes in population demographics, including elevated numbers of immunocompromised patients, elderly adults and premature newborns, as well as the increasing use of inserted foreign bodies, have been reported to contribute to the large variety of CoNS infections [7]. In clinical settings, CoNS have rarely been classified at the species level. Clinical isolates of staphylococci are routinely differentiated between the highly virulent species *Staphylococcus aureus*, which produces coagulase, and coagulase-negative CoNS. Thus, the real impact of minor isolated species of CoNS might be underreported [7]. Moreover, detailed classification of CoNS to species levels has revealed that less frequently isolated CoNS, which are considered less virulent or of animal origin, could cause infections if the patients have underlying conditions such as indwelling foreign bodies and/or immunosuppression [8].

*Staphylococcus caprae* is one of the animal-associated CoNS that usually colonizes skin and mammary glands of goats and sometimes causes goat mastitis [9]. *S. caprae* also causes zoonosis whereby *S. caprae* have been isolated from patients in close contact with goats/sheep, such as farm workers, sheep breeders and those who had been bitten by a goat [10]. However, *S. caprae* infections have recently been recognized as hospital-acquired infections since most *S. caprae* infections were related to medical care and were contracted in hospitals [10]. Reported *S. caprae* infections include bacteremia, acute otitis externa, bone and joint infections, and prosthetic infections [10–14]. However, the reasons why bacteria of animal origin cause nosocomial infections are not well understood.

Phylogenetic analysis using multilocus DNA sequence data (16S rRNA gene, *dnaJ*, *rpoB*, and *tuf* gene fragments) estimated that *S. caprae* belongs to the “Epidermidis cluster group,” which includes *S. epidermidis*, *S. capitis* subsp. *capitis*, *S. capitis* subsp. *urealyticus*, and *S. saccharolyticus* [15]. *S. epidermidis* is the most frequently isolated staphylococcal species from a wide range of human skin niches such as head, legs, and arms [7]. It is also known as one of the most common bacterial pathogens circulating in hospital settings and is the leading cause of nosocomial bloodstream and cardiovascular infections as well as infections associated with implanted medical devices [7, 16]. Since *S. epidermidis*, *S. capitis*, and *S. caprae* all belong to the epidermidis cluster group and cause hospital-acquired bloodstream and implant-associated infections, they are assumed to share the fundamental mechanisms responsible for the various nosocomial infections.

In this study, we determined the complete whole-genome sequences of three *S. caprae* strains isolated from patients with bloodstream or graft infections, or a nasal swab culture of an MRSA screening at hospital admission. To date, genome sequence data from more than 400 *S. epidermidis*, *S. capitis*, and *S. caprae* isolates are available in GenBank. However, since most of these genome sequences are draft genomes generated by de novo assembly of short reads, repetitive sequences are not completely determined. Therefore, we thought that complete genome sequence data are essential for the analysis of genes containing repeat regions, such as the gene encoding a surface adhesin of *S. epidermidis* (fibrinogen-binding protein Fbe), which plays an important role in biofilm formation and devise-related infections.

## Results and discussion

### General features of *S. caprae* genomes

**Fig. 1:**
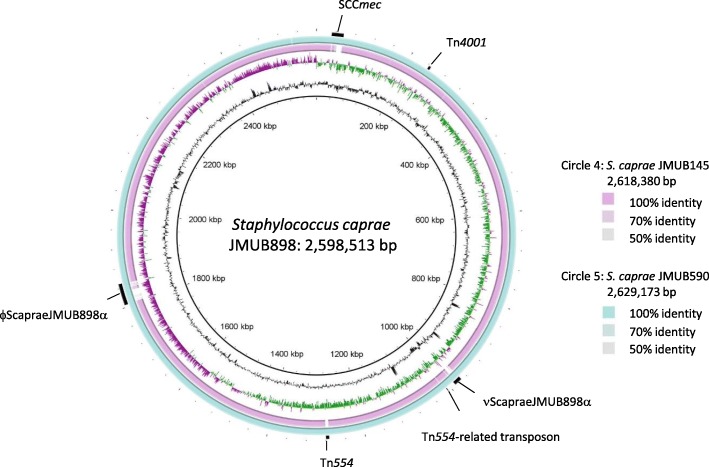
Circular representation of the genome comparison of three *Staphylococcus caprae* strains. Circle 1 (innermost circle) and circle 7 (outermost circle) indicate the distances from the putative origin of replication of the *S. caprae* JMUB898 chromosome. Circles 2 and 3 designate the GC content (black) and GC skew (green, plus strand; purple, minus strand). Circle 4 shows nucleotide identities between *S. caprae* JMUB145 and JMUB898 chromosomes calculated by blastn (light blue). Circle 5 represents nucleotide identities between *S. caprae* JMUB590 and JMUB898 chromosomes. Circle 6 shows prophages, genomic islands, and transposons (black). The *S. caprae* genomes are compared and visualized by BLAST Ring Image Generator v0.95 [19]

We determined complete whole-genome sequences of three methicillin-resistant *S. caprae* strains isolated from humans. Two draft genome sequences of *S. caprae* 9557 and M23864:W1 strains were recently determined, and genome comparison analysis of *S. epidermidis*, *S. capitis*, and *S. caprae* was carried out using two complete genomes of *S. epidermidis* and *S. capitis* and two draft genomes of *S. capitis* and *S. caprae* [17, 18]. However, it is difficult to elucidate the complete features of *Staphylococcus* chromosome structures and genes containing repeats with draft genome sequence analysis. For example, the draft genome sequence of *S. caprae* M23864:W1 strain could not clarify the complete sequences of biofilm-associated genes encoding Embp/EbphA and ClfB-like genes since they might contain repeat sequences. In the case of the draft sequence, assembly is incomplete; there may be errors in the sequences; and annotation is incomplete. Determined complete whole-genome sequences of three methicillin-resistant *S. caprae* strains are shown in Fig. 1. The strains of *S. caprae* JMUB145, JMUB590, and JMUB898 contained one single circular chromosomes, and one, five, and seven plasmids, respectively. The respective chromosome sizes of *S. caprae* JMUB145, JMUB590, and JMUB898 were 2,618,380, 2,629,173, and 2,598,513 bp, with almost identical G + C content (33.6%). A total of 2,489, 2,510, and 2,476 protein-CDSs were annotated on the chromosomes of *S. caprae* JMUB145, JMUB590, and JMUB898, respectively. *S. caprae* JMUB145, JMUB590, and JMUB898 strains were also found to contain six, five, and five rRNA-encoding gene (rDNA) clusters, as well as 59, 59, and 58 tRNA genes, respectively.

**Fig. 2:**
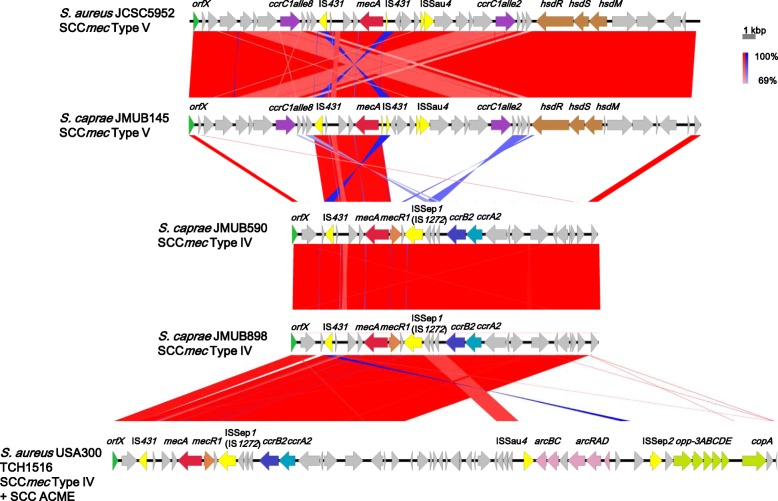
Structure of SCC*mec* in methicillin-resistant *Staphylococcus caprae* strains. The SCC*mec* elements in *S. caprae* strains JMUB145, JMUB590, and JMUB898 were compared with the most related SCC*mec* elements in *S. aureus* JCSC5952 and *S. aureus* USA300 TCH1516 (DDBJ/EMBL/GenBank accession numbers AB478780 and CP000730) using Easyfig 2.2.2 [90]. Arrows and arrowheads indicate CDS (green, *orfX*; light blue, *ccrA*; blue, *ccrB*; purple, *ccrC*; yellow, insertion sequence; red, *mecA*; orange, *mecR1*; brown, restriction system; pink, arginine catabolic gene; light green, oligopeptide permease gene). The blastn matches range from 100% (darkest) to 69% (lightest) and normal and inverted blastn matches shows in blue and red

Figure 1 shows the blastn identities of CDSs among individual genomes of *S. caprae* JMUB898, JMUB145, and JMUB590 chromosomes, with JMUB898 as the central reference chromosomal sequence [19]. Most of the differences between *S. caprae* genomes were found on genomic islands including SCC*mec*, prophages, and transposons (Fig. 1). JMUB145 carried a type V SCC*mec* element. The entire structure of SCC*mec* in the JMUB145 genome was almost identical with that of *S. aureus* JCSC5952, which was isolated from children with impetigo in Japan in 2002 (Fig. 2) [20]. The SCC*mec* of JMUB145 also showed high similarity with that of *S. pseudointermedius* 06–3228, which was isolated from a dog [21]. The SCC*mec* of JMUB145 possessed two *ccrC* genes: *ccrC1* allele 2 (*ccrC2*) and *ccrC1* allele 8 (*ccrC8*). Both JMUB590 and JMUB898 carried type IVa SCC*mec* elements, which are closely related to type IVa SCC*mec* of the *S. aureus* USA300 TCH1516 strain (Fig. 2) [22].

### Phylogenetic relationship among *S. epidermidis*, *S. capitis* and *S. caprae*

**Fig. 3:**
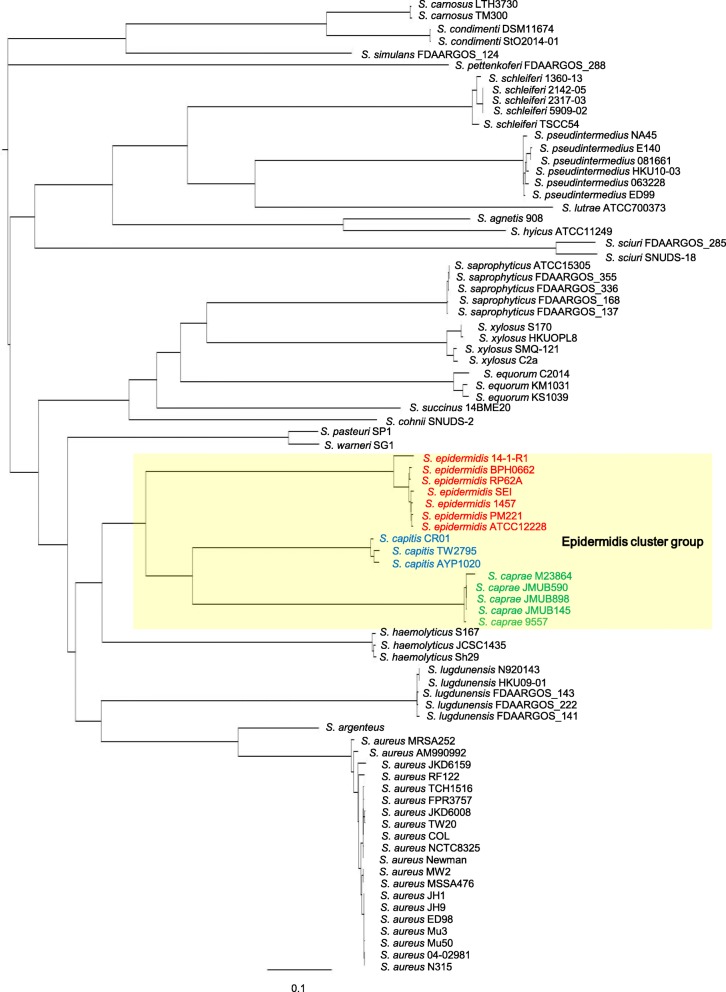
Phylogenetic tree for 24 *Staphylococcus* species. The maximum parsimony tree was constructed using the majority of SNPs present in at least 75% of genomes. The 80 complete genome sequences analyzed were composed of 24 *Staphylococcus* species, and two *S. caprae* draft genome sequences were also included in this analysis (Additional file 1 TableS1)

To date, the genus *Staphylococcus* has been classified into more than 47 species and 23 subspecies, comprising 15 cluster groups, based on a phylogenetic analysis performed using DNA sequence data from multiple loci, such as the 16S rRNA gene and *dnaJ*, *rpoB*, and *tuf* gene fragments [7, 15]. *S. caprae*, together with *S. epidermidis*, *S. capitis* subsp. *capitis*, *S. capitis* subsp. *urealyticus*, and *S. saccharolyticus*, belongs to the epidermidis cluster group [7, 15]. Species of the epidermidis cluster group showed oxidase-negative, novobiocin-susceptible, and coagulase-negative phenotypes. To confirm the phylogeny of the genus *Staphylococcus*, we constructed a genome-wide phylogenetic SNP tree using three *S. caprae* chromosome sequences determined in this study and 80 complete genome sequences of 24 *Staphylococcus* species available on GenBank (Additional file 1 Table S1). We also included two *S. caprae* draft genome sequences, which are also available on GenBank, into the analysis. The phylogenetic analysis confirmed that *S. epidermidis*, *S. capitis*, and *S. caprae* strains of the epidermidis cluster group fell into a single clade (Fig. 3). Based on the SNP tree, the epidermidis cluster group is most closely related to *S. haemolyticus* strains (Fig. 3).

**Fig. 4:**
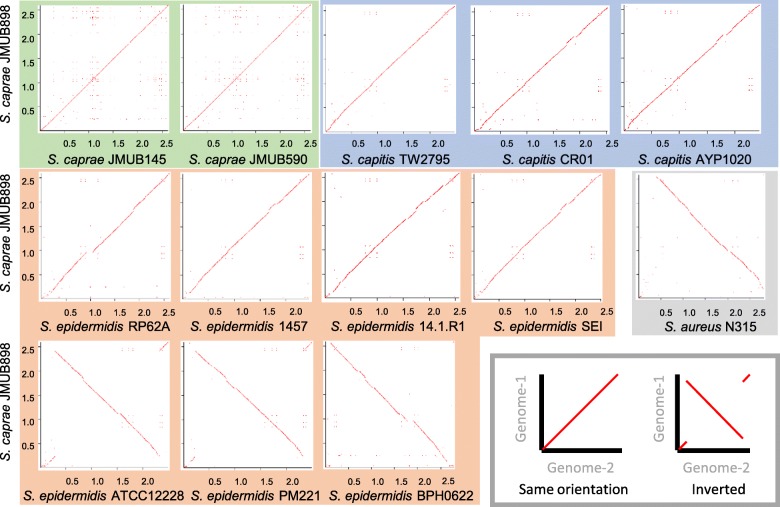
Dot plots of *Staphylococcus epidermidis*, *S. capitis*, and *S. caprae* chromosomes. The *S. caprae* JMUB898 chromosome was compared with those of two *S. caprae* strains (green), three *S. capitis* strains (blue), seven *S. epidermidis* strains (orange), and one *S. aureus* strain (gray) by in silico Molecular Cloning Genomics Edition ver. 6.0.11D. A dot is plotted at every co-ordinates having similarity between two chromosomes

To elucidate the genome structure of the epidermidis cluster group, we compared the entire chromosome sequences among *S. epidermidis*, *S. capitis*, and *S. caprae* strains. The chromosome sequence of *S. caprae* JMUB898 was compared with those of *S. caprae* JMUB145 and JMUB590 by dot plot analysis (Fig. 4). Moreover, the *S. caprae* JMUB898 chromosome sequence was also compared with the complete genome sequences or complete chromosome sequences of seven *S. epidermidis* and three *S. capitis* strains. Dot plot analysis revealed that genome structures were relatively conserved among *S. caprae* and *S. capitis* strains (Fig. 4). However, as compared with the *S. caprae* JMUB898 chromosome, three of seven *S. epidermidis* chromosomes (ATCC12228, PM221 and BPH0622) were inverted around *oriC*. The *S. aureus* chromosome was also inverted around *oriC* when chromosome sequences were compared between *S. caprae* JMUB898 and *S. aureus* N315 (Fig. 4).

### Identification of species-specific genes in *S. epidermidis*, *S. capitis* and *S. caprae* and conserved genes among the epidermidis cluster group

**Fig. 5:**
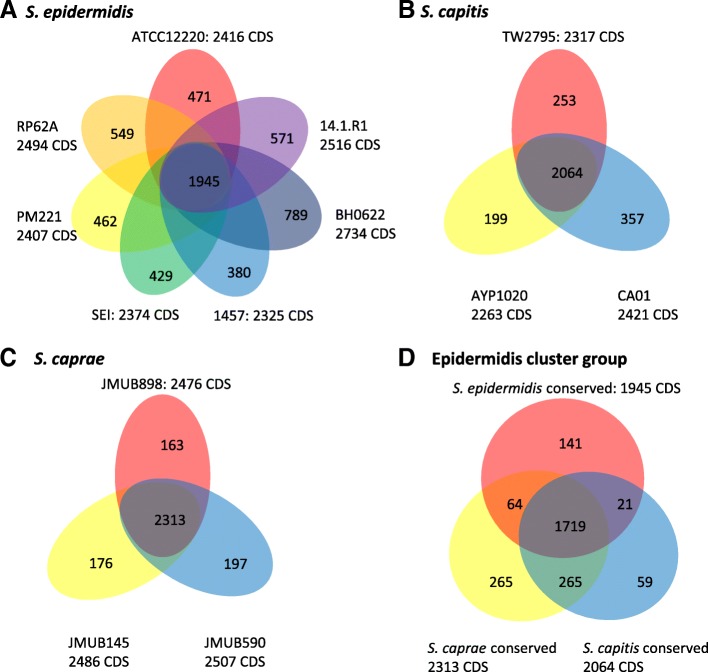
Venn diagram displaying the comparative gene orthology among **a** *Staphylococcus epidermidis*, **b** *S. capitis*, **c** *S. caprae*, and **d** the epidermidis cluster group strains. The numbers indicate the unique genes in each strain or the shared genes between groups of strains, satisfying the criteria of more than 50% amino acid identity on 50% of the total length of a protein

In order to uncover the genetic diversity of the epidermidis cluster group, we first determined the core/pan-genome of each species to estimate the number of shared genes between every *S. epidermidis*, *S. capitis*, and *S. caprae* strain. Although more than 400 genome sequences of the epidermidis cluster group are available on GenBank, including draft genome sequences, we focused on the complete genome-sequenced strains. Complete genome sequences are considered to be more reliable and allow identification of precise strain-specific genes on each chromosome. In this core/pan-genome analysis, genes harbored on genomic islands, prophages, SCC*mec*, and transposons were separated from the category of conserved genes in each species. There were a total of 1,945 conserved genes identified among all *S. epidermidis* strains (Fig. 5 and Additional file 2 TableS2). Similar to *S. epidermidis*, 2,064 and 2,313 conserved genes were shared between every *S. capitis* and *S. caprae* strain, respectively. The conserved genes showed high sequence similarity among the three species. More than 95% of conserved genes displayed > 95% sequence identities by interspecies comparison (Additional file 2 TableS2).

Next, we sought to identify both conserved and species-specific gene sets of the epidermidis cluster group using the conserved gene sets of each species. As shown in Fig. 5, 1,719 CDSs were conserved among the three species, while 141, 59, and 263 species-specific genes were identified in *S. epidermidis*, *S. capitis*, and *S. caprae*, respectively. We also found that 21, 265, and 64 genes were shared between species when comparing *S. epidermidis* vs *S. capitis*, *S. capitis* vs *S. caprae*, and *S. caprae* vs *S. epidermidis*, respectively (Fig. 5d).

**Fig. 6:**
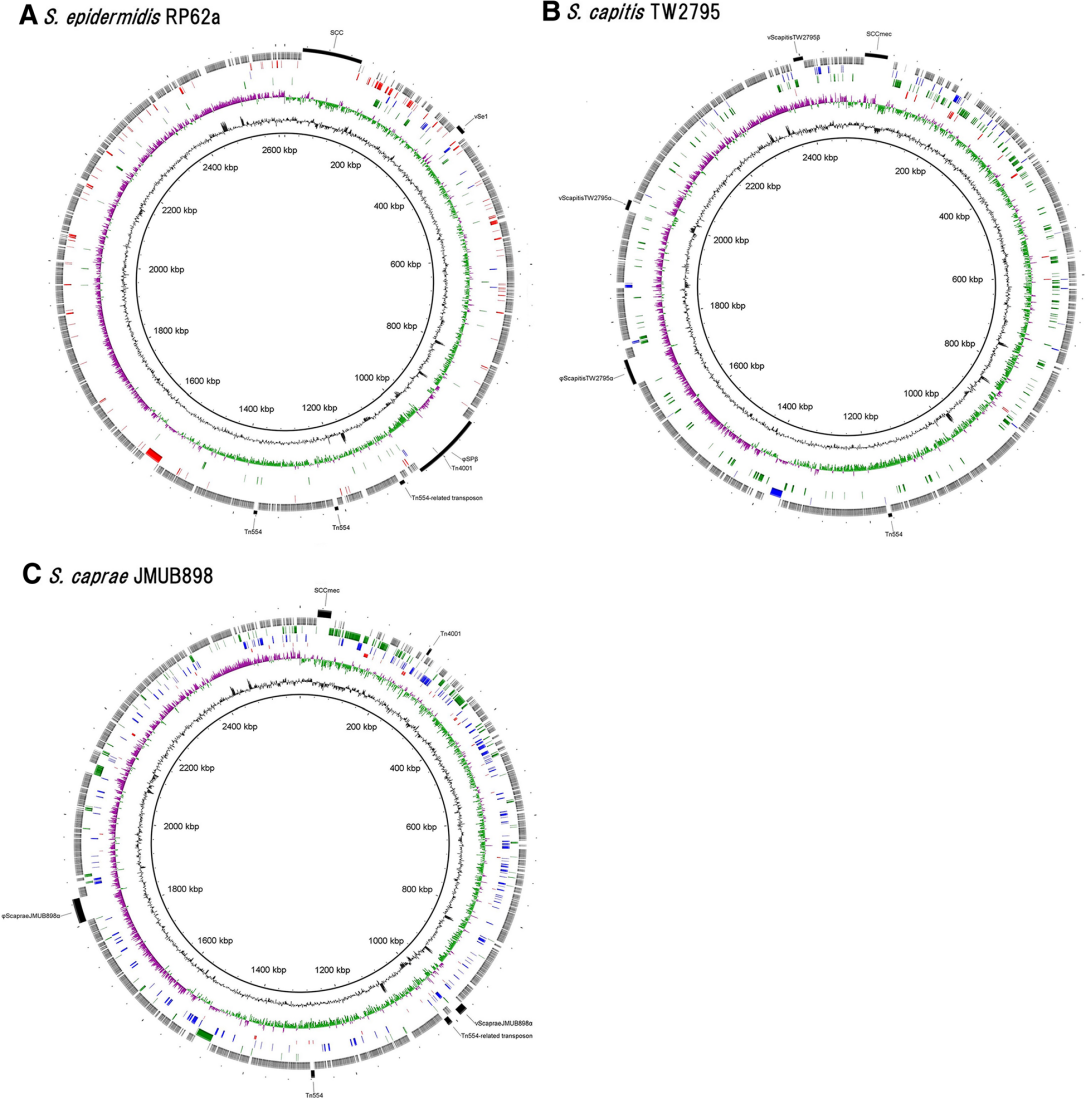
Distribution of core and unique gene sets on the chromosomes of *Staphylococcus epidermidis* RP62a, *S. capitis* TW2795, and *S. caprae* JMUB898. Core and unique gene sets are plotted on the chromosomes of (A) *S. epidermidis* RP62a, (from the periphery toward the center) Circle 1, genomic island; Circle 2, Epidermidis cluster group-conserved genes; Circle 3, *S. epidermidis*-conserved genes; Circle 4, *S. epidermidis*-*S. capitis*-shared gene; Circle 5, *S. epidermidis*-*S. caprae*-shared gene; Circle 6, GC skew; Circle 7, GC count; Circle 8, nucleotide position (B) *S. capitis* TW2795, Circle 1, genomic island; Circle 2, Epidermidis cluster group-conserved genes; Circle 3, *S. capitis*-conserved genes; Circle 4, *S. capitis*-*S. caprae*-shared gene; Circle 5, *S. capitis*-*S. epidermidis*-shared gene; Circle 6, GC skew; Circle 7, GC count; Circle 8, nucleotide position, and (C) *S. caprae* JMUB898, Circle 1, genomic island; Circle 2, Epidermidis cluster group-conserved genes; Circle 3, *S. caprae*-conserved genes; Circle 4, *S. caprae*-*S. capitis*-shared gene; Circle 5, *S. caprae*-*S. epidermidis*-shared gene; Circle 6, GC skew; Circle 7, GC count; Circle 8, nucleotide position, by BLAST Ring Image Generator v0.95 [19]

To analyze the genome robustness or plasticity of the epidermidis cluster group, the gene sets specific to each species, shared between two species, and conserved among all three species were mapped on *S. epidermidis* RP62a, *S. capitis* TW2795, and *S. caprae* JMUB898 chromosomes (Fig. 6). The gene synteny was found to be well retained in the chromosome of the epidermidis cluster group. However, the chromosomal regions where some species-specific genes were clustered in each species seem to be the remnants of genomic islands.

The downstream regions of SCC*mec* elements also were not conserved among the three species. Our analysis revealed that the species-specific genes of *S. capitis* and *S. caprae* were typically mapped to the downstream of SCC elements, similar to that of *S. aureus*, *S. epidermidis*, and *S. haemolyticus* genomes [23] (Fig. 6). Some remnants of prophages or genomic islands were also found (Fig. 6). These species-specific genomic regions are considered likely to contribute to the evolution and differentiation of *Staphylococcus* species differentiation [23].

Although the downstream regions of the SCC*mec* elements were divergent among species, we found that the type V SCC*mec* identified in the *S. caprae* JMUB145 genome was closely related to those of *S. aureus* JCSC5952 and *S. pseudointermidius* 06–3228 (Fig. 2) [20, 21], while type IVa SCC*mec* of *S. caprae* JMUB590 and JMUB898 was closely related to that of the *S. aureus* USA300 TCH1516 strain (Fig. 2) [22]. The SCC*mec* of methicillin-resistant *S. caprae* has rarely been analyzed. Previous studies showed that *S. caprae* stains carried a variety of *mec* gene complexes, for example, one *S. caprae* strain isolated from pig was reported to carry class A *mec* and type I *ccr* [24, 25], and in *S. epidermidis* and *S. capitis* strains, type I–V SCC*mec* elements (originally identified in MRSA) were identified by PCR-based methods [7]. It might be interpreted as the results of co-evolution and interspecies exchange of the SCC*mec* elements over long-term evolution.

### Genes conserved across three species

Our analysis identified 1,719 conserved CDSs among *S. epidermidis*, *S. capitis*, and *S. caprae*. Most of the conserved genes are involved in fundamental biological processes. Compared with the highly virulent *S. aureus*, the three species possessed fewer known and putative virulence factors. Well-known virulence factors of *S. aureus* such as coagulase, protein A, leukocidins, α-toxin, and staphylococcal enterotoxins were missing from these genomes. Nevertheless, the three species shared virulence factors involved in biofilm formation and protection against the innate immune system. Furthermore, teichoic acid biosynthesis genes (*tagAHGBXD*) encoding wall teichoic acid (WTA) were conserved among the three species. This gene cluster confers positive charge to bacterial cells, thereby mediating primary adherence to polystyrene surfaces as well as host organisms to initiate biofilm formation [7]. The *dltABCD* genes were also conserved among the three species. *dltABCD* genes function as the modification system for WTA by incorporating d-alanine into WTA [26]. Our genome analysis also confirmed that all analyzed genomes from the three species (*S. epidermidis*, *S. capitis*, and *S. caprae*) shared poly-γ-dl-glutamic acid (PGA) genes. The PGA capsule is another *Staphylococcus* extracellular virulence factor found in *S. epidermidis*, which facilitates bacterial growth and survival in the human host [27]. Kocinaova et al. described that all tested *S. epidermidis* strains and the reference strains of the epidermidis cluster group, including *S. capitis* subsp. *capitis*, *S. capitis* subsp. *ureolyticus*, and *S. caprae*, produced PGA, and carried *capB* and *capD* genes [27]. The other extracellular proteins such as secreted thermonuclease [28] and Clp protease [29] involved in biofilm formation were also found to be conserved among the three species.

The three *S. caprae* strains carried two phenol soluble moduline (PSM) gene clusters, PSMβ and PSMα/PSMδ. The former was located upstream of Arginine-tRNA and was conserved among the *Staphylococcus* species, including *S. epidermidis, S. capitis*, and *S. caprae*. The number of genes of a PSMβ cluster has been shown to vary from two to six [17]. Consistent with this previous report [17], the PSMβ cluster of the three *S. caprae* strains contained five genes, while that of *S. epidermidis* RP62a and *S. capitis* TW2795 consisted of four genes. The PSMα/PSMδ cluster of *S. caprae* was located upstream of the NADH dehydrogenase gene and contained single copies of PSMα and PSMδ. PSMα and PSMδ were conserved in *S. epidermidis* RP62a and *S. capitis* TW2795 strains.

### Divergent interspecies evolution of cell surface extracellular proteins

Cell surface extracellular proteins mediate bacterial adherence to abiotic surfaces and host tissues as well as intercellular adhesion during biofilm formation [7]. By analyzing the conserved genes among the three species, *S. epidermidis*, *S. capitis*, and *S. caprae*, we found that most cell surface proteins showed substantially lower sequence identities between species compared with the other conserved proteins. The average of amino acid identities for total conserved genes were 80.4, 90.9, and 84.8% among *S. epidermidis* RP62a vs *S. capitis* TW2795, *S. capitis* TW2795 vs *S. caprae* JMUB898, and *S. caprae* JMUB898 vs *S. epidermidis* RP62a, respectively. In contrast, the identities for cell surface proteins were less than 50%. Since we set a cutoff value of 50% for amino acid identity in this analysis, proteins with amino acid identities of less than 50% must be categorized as species-specific proteins. However, even though most cell surface proteins had less than 50% identities among the three species, they could be considered as homologues of each other, because they were judged to share the same ancestors or origin by analyzing gene structure, location on the genome, and functional motifs. For example, *S. capitis* and *S. caprae* carried SesC-like proteins. The amino acid sequence identities of SesC/SesC-like proteins were 45.0% between *S. epidermidis* RP62a and *S. capitis* TW2795 and 46.1% between *S. epidermidis* RP62a and *S. caprae* JMUB898. The SesC-like proteins are postulated to have a similar function as SesC in *S. epidermidis* because SesC-like proteins in *S. capitis* and *S. caprae* contain the LPXTG motif, as does SesC in *S. epidermidis*. Moreover, the genome loci of the genes were considered to be the same since the upstream region of every *sesC* in these three species contained a gene encoding an organic hydroperoxide resistance-like protein. Therefore, SesC-like proteins in *S. capitis* and *S. caprae* might function as host cell factors and/or abiotic surface-binding proteins owing to the fact that SesC in *S. epidermidis* is one of the fibrinogen-binding proteins containing a wall-anchoring LPXTG motif that mediates biofilm formation [30] although the amino acid identities of the three proteins were shown to be less than 50%. These lower identities seemed to reflect their divergent interspecies evolution.

**Fig. 7:**
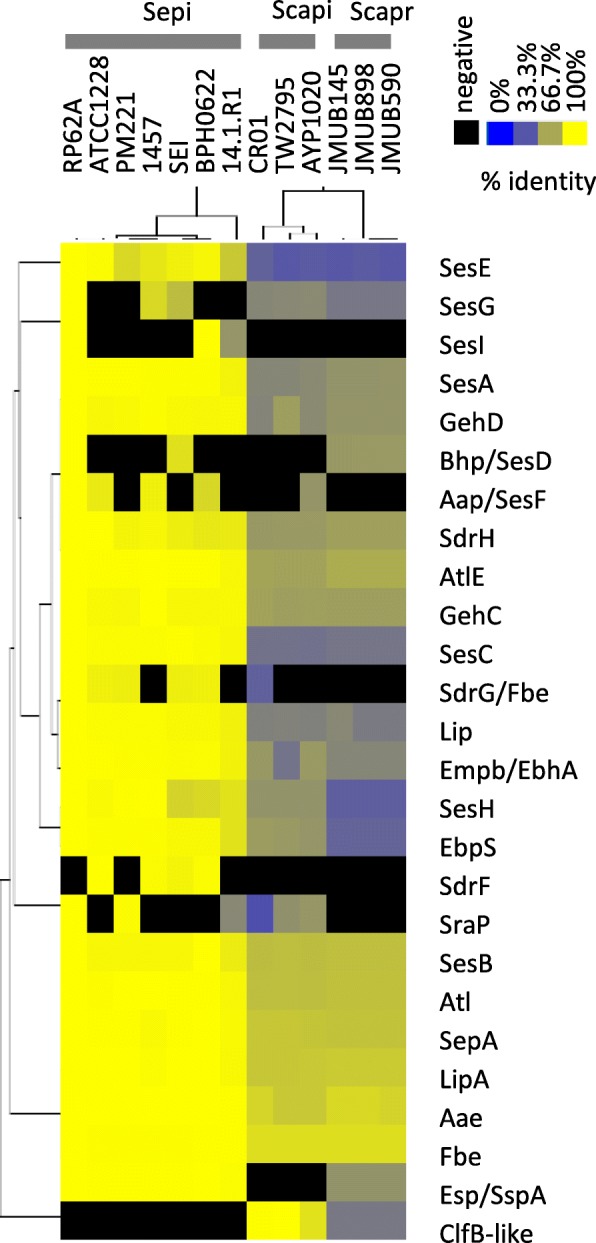
Heat map of amino acid identities of cell wall-anchored proteins among *Staphylococcus epidermidis*, *S. capitis*, and *S. caprae* strains. A heat map was generated using pairwise identity matrix tables with hierarchical clustering method calculated by Cluster 3.0. The heat map was visualized by Java Treeview 1.1.6r4

In this study, we also identified the cell surface proteins involved in adhesion and biofilm formation and created a heat map of these proteins based on their amino acid identities (Fig. 7). We found that 17 of the 26 cell surface proteins were well conserved in the three species. Eleven cell-wall-anchored proteins, SesA-I and SdrFG, which contained an N-terminal secretion signal sequence and wall-anchoring LPXTG motif, were predicted from the *S. epidermidis* RP62a genome sequence [31]. These proteins are involved in bacterial attachment to host tissue or cells and biofilm formation. Although 5 (*sesABCEH*) of the 11 genes encoding the cell wall-anchored proteins were identified at the similar genome locus tags of the three species, there were few interspecies similarities. The amino acid identities of SesA, SesB, SesC, SesE, and SesG between *S. epidermidis* RP62A and *S. capitis* TW2795 were 53.2, 75.0, 45.0, 34.6, and 58.0%, respectively, while those between *S. epidermidis* RP62A and *S. caprae* JMUB898 were 58.3, 75.5, 46.1, 35.9, and 37.2%, respectively. In addition to SesA-I and SdrFG, *S. epidermidis* strains carry an extracellular matrix-binding protein Embp/EbhA that is similar to Ebh identified in *S. aureus* [32, 33]. *S. capitis* and *S. caprae* also carry Embp/EbhA-like proteins with the amino acid identities of ~ 60.3% compared with those of *S. epidermidis*.

*S. epidermidis* carries three multifunctional autolysin/adhesins, AtlE, Atl, and Aae, which mediate both cell lysis and attachment to materials including host tissue or cells, abiotic surfaces, and extracellular DNA [27, 34]. Similar to other cell surface proteins containing an LPXTG motif, these proteins are conserved among the three species, but the amino acid identities were less than the average amino acid identities of conserved proteins. The most analyzed staphylococcus autolysin is AtlE in *S. epidermidis,* and the *atlE* gene disseminates among staphylococcal species. AtlE homologues, such as Atl in *S. aureus*, Atl_wm_ in *S. warneri*, AtlC in *S. caprae*, Aas in *S. saprophyticus*, and AtlL in *S. lugdunensis*, have been reported as fibronectin-binding proteins involving in biofilm formation [35–37].

Most organisms colonizing human skin possess lipolytic activity to hydrolyze lipids found on the surface of human skin [38]. Other than cell wall-anchored proteins, extracellular lipases, GehCD, Lip, and LipA, which mediate bacterial colonization on human and animal skins, were conserved in *S. epidermidis*, *S. capitis*, and *S. caprae* with high interspecies diversity [39, 40]. Every strain in the epidermidis cluster group also possesses an extracellular metalloprotease with elastase activity, such as SepA (SE2219) that plays a role in conferring resistance to the antimicrobial peptide dermicidin [41].

The elastin-binding protein (*ebpS*) or *ebpS*-like genes were also found to be conserved among *S. epidermidis, S. capitis*, and *S. caprae* strains. Similar to extracellular lipases, EbpS also showed high diversity among the three species. Our analysis categorized *S. epidermidis* EbpS as a different protein from the EbpS of *S. capitis* and *S. caprae* since the amino acid identity of EbpS proteins between *S. epidermidis* and *S. caprae* was less than 39.9%, and the length of the homologous region between EbpS of *S. epidermidis* and *S. capitis* was less than 50% of the entire region. As reported in *S. aureus*, EbpS was first identified as an adhesin for extracellular matrix elastin of host cell tissue [42]. However, EbpS in *S. aureus* shows relatively weak binding potential to elastin [43]. The adherence of some *S. aureus* strains to immobilized elastin is mediated by fibronectin-binding proteins FnBPA and FnBPB, but not by EbpS, and the inactivation of *ebpS* in *S. aureus* strains has only a minimal effect on the binding of *S. aureus* to elastin peptide [43]. Regardless, it was recently reported that EbpS regulates bacterial growth rate in liquid culture and promotes biofilm maturation in a zinc concentration-dependent manner [44]. Therefore, EbpS and EbpS-like proteins of the epidermidis cluster group might play a role in biofilm maturation.

### Genes shared by *S. capitis* and *S. caprae* but not by *S. epidermidis*

Based on the phylogenetic analysis, *S. capitis* is more closely related to *S. caprae* than *S. epidermidis*. Therefore, a larger set of shared genes could be identified between *S. capitis* and *S. caprae* than when comparing *S. epidermidis* with *S. capitis* or *S. epidermidis* with *S. caprae* (Fig. 5 and Additional file 2 TableS2). Four major biofilm formation-related factors were shared by *S. capitis* and *S. caprae***.** These include polysaccharide intracellular adhesin (PIA), elastin-binding protein (EbpS), SesC-like proteins, and SdrH-like proteins. Since EbpS, SesC, and SdrH of *S. capitis* and *S. caprae* (EbpS-like, SesC-like, and SdrH-like proteins) showed lower similarities (add number %) to those of *S. epidermidis*, they were categorized as shared by *S. capitis* and *S. caprae.*

PIA, also known as poly-*N*-acetylglucosamine, mediates biofilm formation and plays an important role in immune evasion [45–47]. PIA production is regulated by the *ica* operon (*icaADBC*) [45, 48], and the *ica* locus has been identified in many staphylococcal species including *S. aureus*, *S. capitis*, and *S. caprae* [49, 50]. We found that all *S. capitis* and *S. caprae* strains analyzed in this study possessed the *ica* operon. However, only three *S. epidermidis* strains (RP62a, 1457, and BH0622) carried the *ica* operon. This is in concordance with a previous study where a number of *ica* negative clinical isolates of *S. epidermidis* were reported [51].

SesG is one of the cell surface–anchored proteins identified in the *S. epidermidis* RP62a genome. Although four of the seven *S. epidermidis* strains lack the *sesG* gene, every *S. capitis* and *S. caprae* strain analyzed in this study carried the *sesG* gene, which had amino acid identities of 53.2–54.6% (*S. epidermidis* RP62a vs *S. capitis*) and 47.2–53.2% (*S. epidermidis* RP62a vs *S. caprae*).

*S. capitis* and *S. caprae* also shared the same mannitol metabolic pathways. Mannitol utilization has been adapted for species classification among CoNS [52]. Our genome analysis confirmed that mannitol acquisition and utilization pathways are conserved in mannitol-positive species such as *S. capitis* and *S. caprae*, while mannitol-negative *S. epidermidis* species lacks this system. Arginase, which catalyzes the fifth and final steps in the urea cycle, resulting in the conversion of l-arginine into l-ornithine and urea, was conserved in *S. capitis* and *S. caprae* but not in *S. epidermidis*. It is interesting that urease genes were conserved in all three species, yet urease activity, being one of the key phenotypes for classification of CoNS, was shown to be negative in *S. capitis* subsp. *capitis* [52]. *S. capitis* and *S. caprae* also shared staphyloxanthin biosynthesis genes *crtOPQMN*. These genes regulate the production of orange carotenoid, conferring the characteristic golden-yellow color of colonies, which aids in distinguishing *S. aureus* from *S. epidermidis* [53]. A clumping factor B-like protein gene, located at the identical locus downstream of the *arcR* gene was identified in *S. capitis* and *S. caprae* genomes but not in the *S. epidermidis* genome.

### Genes shared by *S. epidermidis* and *S. capitis* but not by *S. caprae*

*S. epidermidis* and *S. capitis* shared 21 genes (Fig. 5), among which are the accessory Sec systems. The canonical Sec system, which translocates the majority of proteins across the cytoplasmic membrane, is present in all bacteria including staphylococcal species. In addition to the canonical Sec system, accessory Sec systems were found in *S. epidermidis* and *S. capitis* but not in *S. caprae.* The accessory Sec systems are conserved in staphylococci and streptococci, facilitating the transportation of serine-rich repeat glycoproteins [54, 55]. In *S. aureus*, the accessory Sec system secretes the serine-rich glycoprotein SraP, which mediates staphylococcal binding on human platelets [54]. *S. capitis* possesses a SraP homologue (JMUB0001_172). Although the SraP homologue was not conserved in all *S. epidermidis* strains, we identified it in *S. epidermidis* RP62a, PM221, and 14.1.R1 strains. This finding indicated that the additional Sec system might contribute to glycoprotein translocation.

Our genome comparison analysis showed that 5 of the 11 cell wall-anchored proteins identified in the *S. epidermidis* RP62a genome were conserved among *S. epidermidis*, *S. capitis*, and *S. caprae* (Fig. 7 and Additional file 2 TableS2). Another two genes, *sesF* and *sdrG*, were identified only in some *S. epidermidis* and *S. capitis* strains but not in *S. caprae*. The *sesF* gene, encoding the accumulation-associated protein Aap, had been identified as an essential protein for biofilm accumulation on glass or polystyrene surfaces in certain *S. epidermidis* strains [56, 57]. The giant extracellular protein mediates biofilm formation and accumulation via fibronectin-binding and intercellular adhesion abilities [58, 59]. Four of the seven genome-sequenced *S. epidermidis* strains and one of the three *S. capitis* strains carried the *aap* gene on their chromosomes. In contrast, five of the seven genome-sequenced *S. epidermidis* strains carried the *sdrG*/*fbe* gene, while 1457 and 14.1.R1 lacked this gene. *S. capitis* CR01 also had the SdrG/Fbe-like protein with an amino acid identity of 38.4% between RP62a and CR01. SdrG, one of the three SD repeats-containing serine asparagine-rich (Sdr) proteins identified in *S. epidermidis* RP62a (also known as fibrinogen-binding protein [Fbe]), was suggested to promote device-related infection due to the observation of that an *sdrG*/*fbe* deletion mutant was attenuated in an intravascular catheter-associated rat infection model [60–62].

### Genes shared by *S. epidermidis* and *S. caprae* but not by S. capitis

We found that *S. epidermidis* shared 64 genes with *S. caprae* strains (Fig. 5). Every sequenced *S. epidermidis* and *S. caprae* strain carried serine V8 protease GluSE, also known as Esp/SspA (SE1543) [63–65]. When *S. epidermidis* and *S. aureus* co-exist in an organism, Esp produced by commensal *S. epidermidis* strains inhibits *S. aureus* biofilm formation and subsequently its colonization in the anterior nares [66]. The *esp* gene was not found in the three sequenced *S. capitis* strains.

All sequenced *S. epidermidis* and *S. caprae* strains also carried the arginine synthesis pathway genes *argJBCD*. l-Arginine is required for the growth of most *Staphylococcus* species, including *S. aureus*, *S. epidermidis*, and *S. capitis* [67]. The presence of *argJBCD* genes in *S. epidermidis* and *S. caprae* is consistent with a previous study where the mutants not requiring l-arginine were generated by in vitro selection from *S. epidermidis* strains but not from *S. capitis* strains [67]. This implies that *S. epidermidis* strains might have the arginine biosynthesis enzymes, but the enzymes were not expressed in the wild-type strains [67].

### Genes carried by *S. epidermidis* but not by *S. capitis* and *S. caprae*

Each of the seven *S. epidermidis* strains tested carried 380–789 strain-specific genes. The conserved gene analysis identified 141 genes that were conserved in all *S. epidermidis* strains but not in *S. capitis* or *S. caprae*. Most of these *S. epidermidis*-specific genes encode virulence factors that have been known to mediate biofilm formation or be involved in the central metabolic pathways.

The cell wall-anchored protein SdrF was identified only in four of seven *S. epidermidis* strains. The full-length *sdrF* gene, which encodes a type I collagen-binding protein, was identified in SEI, 1457, and BH0622 genomes. Nevertheless, truncated *sdrF* genes were found in *S. epidermidis* RP62a, PM221, and 14.1R1. *S. capitis* AYP1020 also carries the truncated SdrF homologue with 73.3% amino acid identity, but the *S. capitis sdrF* gene was fragmented by a frameshift mutation.

The genome comparison revealed that *S. epidermidis* has a specific central metabolism pathway that does not exist in the genomes of *S. capitis* and *S. caprae*. *S. epidermidis* possesses the glycerol dehydrogenase gene *gldA*, which mediates glycerol metabolism. A previous study showed that *S. epidermidis* could ferment glycerol into succinate, and the resulting succinate could in turn inhibit the growth of another skin colonizer *P. acnes* in vitro and in vivo [68]. Moreover, *S. epidermidis* strains, but not *S. capitis* and *S. caprae* strains, carried fumarate reductase-mediating succinate fermentation and accumulation [69]. *S. epidermidis* also carried biosynthesis genes for biotin, which is a cofactor of acetyl-CoA carboxylase involved in fatty acid metabolism.

### Genes carried by *S. capitis* but not by *S. epidermidis* and *S. caprae*

Each of the three *S. capitis* strains tested carried 199–357 strain-specific genes, in which 59 were identified by our analysis to be *S. capitis* specific. Many species of Gram-positive bacteria, including staphylococci, produce lantibiotics and small cationic antimicrobial peptides to provide antimicrobial activities in facilitating niche compensation [70]. Some CoNS strains produce unique lantibiotics such as epidermin (from *S. epidermidis*) and gallidermin (from *S. Gallinarum*) [71–73]. Kumar et al. reported an identification of an *S. capitis* strain TE8 that carried both epidermin and gallidermin gene loci [17]. In our analysis, all three *S. capitis* strains tested carried lantibiotics genes on their chromosomes, but they were similar to that of *S. aureus* RF122 with an amino acid identity of 47% [74].

All three *S. capitis* strains and *S. epidermidis* RP62A, PM221, and 14.1.R1 carried serine-rich adhesin for platelet (SraP) homologues. SraP homologues have been widely identified among staphylococci including *S. aureus* and *S. gordonii* [75]. SraP mediates bacterial binding to platelets, whereby the binding is considered the key step during infective endocarditis [75, 76].

### Genes carried by *S. caprae* but not by *S. epidermidis* and *S. capitis*

Our genome analysis identified a total of 265 genes that were carried by *S. caprae* but not by any of *S. epidermidis* and *S. capitis* strains, of which 163–197 were carried by five individual strains of *S. caprae* tested. Among those, biofilm-associated protein (Bap), capsular polysaccharide, and type VII secretion factor recognized as virulence factors were included. Bap is known to mediate bacterial attachment to polystyrene and biofilm formation in *S. aureus* and some CoNS [77, 78].

Capsular polysaccharide production is an important virulence determinant in many invasive bacterial pathogens. Capsular polysaccharides are produced by ~ 90% of *S. aureus* strains, and encapsulated *S. aureus* strains are more resistant to phagocytosis than the nonencapsulated strains [79]. Among the CoNS, *S. haemolyticus*, *S. hyicus*, and *S. lentus* produce a capsular polysaccharide-like surface antigen that cross-react serologically with *S. aureus* type 5 capsular polysaccharide [80].

In our genome analysis, three *S. caprae* strains carried a capsular gene operon composed of 16 genes. Even though the first four genes in this operon showed high amino acid identities to *S. aureus cap* 5ABCD, *S. caprae’s* capsular polysaccharide might form a different structure compared with *S. aureus’s* capsular polysaccharide. The *S. caprae cap* operon contained *capHIOM* homologues, and the *cap* operon encoded putative polysaccharide modification enzymes such as glycosyltransferases, *O*-acetyltransferase, and aminotransferase.

The type VII secretion system is encoded by the genomes of diverse bacterial species across the *Firmicutes* and *Actinobacteria* phyla, including *S. aureus* and *Mycobacterium tuberculosis*. The *S. aureus* type VII secretion system exports several effector proteins including EsxABCD and nuclease toxin EsaD, enabling long-term survival in abscesses [81–85]. The gene component of the type VII secretion system cluster varies among *S. aureus* strains, but the gene cluster of *S. caprae* showed high similarity to those of *S. aureus,* e.g. there were 60% identity and 94% similarity of EsaA between *S. caprae* JMUB898 and *S. aureus* MRSA252. The *S. caprae* type VII secretion system contained eight genes encoding four membrane-associated proteins (EsaA, EssAB, and EssC), two soluble cytosolic proteins EsaBC, and two secreted virulence factors EsxAB.

The utilization of trehalose as a carbon source is a key characteristic of *S. caprae* because most *S. epidermidis* and *S. capitis* strains cannot use trehalose as a carbon source [9, 86]. Our genome analysis confirmed that the trehalose-specific PTS system was found only in *S. caprae* genomes but not in *S. epidermidis* and *S. capitis.*

### Biofilm formation capacity of *S. caprae*

**Fig. 8:**
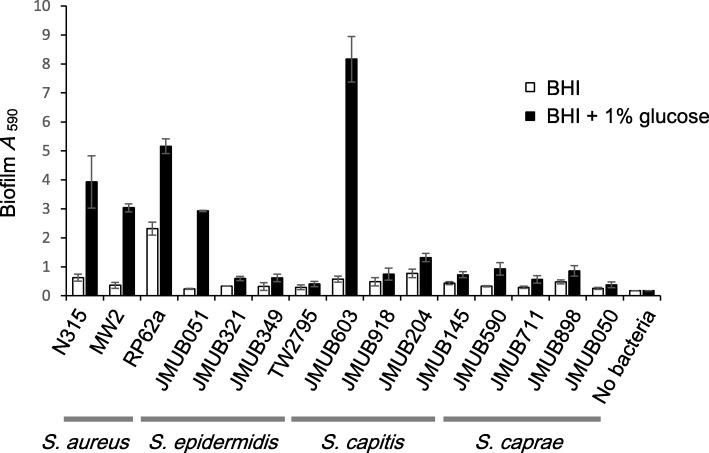
Biofilm formation on a plastic surface. The amount of biofilm on a plastic surface was assessed using crystal violet stain after a 24-h incubation in BHI broth with or without glucose in a plastic titer plate

In order to assess whether *S. caprae* strains can produce biofilms, we carried out biofilm assays in comparison with other staphylococcal species that are well known as biofilm producers. Four *S. epidermidis*, four *S. capitis*, five *S. caprae*, and two *S. aureus* strains were compared (Fig. 8). All strains tested formed biofilm on a plastic surface and the biofilm formation was induced by adding 1% glucose. However, the levels of biofilm formation varied among strains. Two *S. aureus* strains (N315 and MW2), two *S. epidermidis* strains (RP62a and JMUB051), and one *S. capitis* strain (JMUB603) produced higher amounts of biofilm mass on a plastic surface than the other strains. The *S. caprae* strains produced detectable but weaker biofilms.

## Conclusions

Complete whole-genome sequencing and comparative genome analysis revealed that *S. caprae* was highly similar to *S. epidermidis* and *S. capitis.* Moreover, these three coagulase-negative staphylococcal species commonly shared many virulence genes represented by biofilm formation-associated genes, which encode factors that may tolerate having similar pathogenesis features, for example, causing infections associated with indwelling medical devices or catheter use. These virulence factors can be considered as the main characteristics of the epidermidis cluster group. We found that *S. caprae* was differentiated from *S. epidermidis* and *S. capitis* by species-specific virulence factors, capsular polysaccharides and type VII secretion system, which had not been found in *S. epidermidis* and *S. capitis* genomes, but were found in *S. aureus* genomes. The role of these factors on pathogenesis in humans remains largely unknown. It can be speculated that these *S. caprae*-specific factors may confer pathogenicity in animals such as goats.

## Methods

### Bacterial strains

All three methicillin-resistant *S. caprae* strains JMUB145, JMUB898, and JMUB590 were isolated from Jichi Medical University Hospital in Japan in 2015. Both *S. caprae* JMUB145 and JMUB898 are clinical strains; the former was isolated from the blood culture of woman with a fever during the course of chemotherapy treatment for uterine cancer, while the latter was isolated from man with graft infection. In contrast, *S. caprae* JMUB590 was a colonizing isolate obtained from a nasal swab during screening for MRSA colonization of a patient admitted to Jichi Medical University Hospital in 2015. *S. caprae* strains were cultivated at 37 °C in tryptic soy broth (TSB), brain heart infusion (BHI) broth, or on tryptic soy agar (Becton Dickinson Co., Ltd., Sparks, MD). The strains were stored at − 80 °C in 50% glycerol (Wako Pure Chemical Industries, Ltd., Tokyo, Japan). We analyzed biofilm formation of three *S. epidermidis* strains (JMUB051, JMUB321, and JMUB349), three *S. capitis* strains (JMUB603, JMUB918, and JMUB204) and two *S. caprae* strains (JMUB711 and JMUB050), all of which were isolated from patients in Jichi Medical University Hospital between 2015 and 2016. We also performed biofilm assays using genome-sequenced strains: *S. aureus* N315, *S. aureus* MW2, *S. epidermidis* RP62a, and *S. capitis* TW2795.

### DNA extraction

Overnight culture of *S. caprae* strains grown in 10 ml of TSB at 37 °C was harvested by centrifugation with 8,000 rpm for 5 min. The pelleted cells were then resuspended in 3 ml of TE buffer containing 150 μg of lysostaphin (Sigma-Aldrich. Co., LLC., St Louis, MO) and 100 μg of RNase A (Sigma-Aldrich. Co., LLC.). Following a 2-h incubation at 37 °C, 525 μl of 10% SDS and 37.5 μl of Proteinase K solution (Qiagen, Hilden, Germany) were added to the cell lysate. The mixture was then incubated at 56 °C for 2 h. After incubation, one volume of tris-saturated phenol (pH 8.0) was added, and the resulting solution was mixed well by inverting the tube for 5 min. After centrifugation, the aqueous phase was transferred into a new tube. An equal volume of chloroform: isoamylalcohol in the ratio of 24: 1 (vol/vol) was added and mixed well. Following centrifugation, the aqueous phase was transferred into a new tube and a 1/25 volume of 5 M NaCl and 2.5 volume of ethanol were added. After centrifugation, the DNA pellet was washed by 80% ethanol and dried briefly. TE buffer was added to dissolve the DNA. The extracted DNA was treated with RNase again and purified with a DNeasy Blood and Tissue Kit (Qiagen).

### Whole genome sequencing of *S. caprae* strains

Whole-genome sequencing of *S. caprae* was performed as previously described [87]. Briefly, mate-pair sequencing libraries were constructed from genomic DNA of *S. caprae* strains using the Nextera mate-pair sample preparation kit (Illumina, Inc., San Diego, CA, USA) without size selection. Sequencing was performed using the Illumina MiSeq platform (2 × 301 bp) with MiSeq reagent kit version 3 (Illumina, Inc.). The resulting 813,708, 1,058,128, and 1,268,939 paired-end reads of JMUB898, JMUB145, and JMUB590, respectively, were subjected to quality trimming using the FASTQ toolkit version 2.0.0 with quality levels of 35 (JMUB898), 25 (JMUB145), and 35 (JMUB590). A total of 747,050 (JMUB898), 1,057,765 (JMUB145), and 1,168,116 (JMUB590) high-quality reads were then assembled with the Velvet de novo assembly version 1.2.10 algorithm. The generated assemblies composed of 24, 25, and 18 scaffolds, with the longest scaffolds reported to be 2,584,878, 2,606,760, and 2,615,026 bp in JMUB898, JMUB145, and JMUB590, respectively, which are close to the expected size of the *Staphylococcus* genome. Persisting gaps, 18, 23, and 14, in the three respective strains were closed by gap-spanning PCR, followed by Sanger sequencing using an ABI3130xl genetic analyzer (Applied Biosystems, Carlsbad, CA, USA) to generate a single circular genome. The genome sequences were automatically annotated with Microbial Genome Annotation Pipeline (MiGAP, https://www.migap.org/) [88] and manually extracted and annotated.

### Construction of a phylogenetic tree

The phylogenetic tree of staphylococci was constructed using kSNP program version 3.021 [89]. Whole-genome analysis of phylogenies were conducted based on single-nucleotide polymorphisms (SNPs) in the complete whole-genome sequence data of 82 *Staphylococcus* strains including three complete genome sequences and two assembled genomes of *S. caprae* strains (Additional file 1 TableS1). To extract SNPs from sequence data, we applied k-mer 13 as the optimal value predicted by the kSNP-associated Kchooser script. A maximum parsimony tree was constructed using the majority of SNPs present in at least 75% of genomes. The phylogenetic tree was visualized by FigTree ver.1.4.3 (tree.bio.ed.ac.uk/software/figtree/).

### Gene content analysis

The complete genome sequences of seven *S. epidermidis* (14–1-R1, 1457, ATCC12228, BPH0662, PM221, RP62A and SEI), three *S. capitis* (AYP1020, CR01 and TW2795), and three *S. caprae* strains (JMUB145, JMUB590 and JMUB898) (Additional file 1 TableS1) were used for the identification of pan and core genomes. First, coding DNA sequences (CDSs) harbored on the mobile genomic islands such as SCC*mec*, transposons, and prophages were extracted from gene sets of each species and were categorized as accessory genes. The gene sets from paired strains of each species were then compared by pairwise blastp analysis using in silico Molecular Cloning Genomics Edition ver. 6.0.11D (in silico biology, Inc. Yokohama, Japan) and those sharing an amino acid sequence of over 50% identity and over 50% length by the blastp search (Additional file 2 TableS2) were defined as species-conserved genes for the strain. Next, in order to identify the gene set conserved among all three species, pairwise blastp analysis was performed on each species-conserved gene set of *S. epidermidis*, *S. capitis*, and *S. caprae* (Additional file 2 TableS2). Circular representation of *Staphylococcus* chromosomes and whole-genome comparison were performed by BLAST Ring Image Generator v0.95 based on blastn analysis [19]. Pairwise blastn analysis for SCC*mec* regions were carried out using Easyfig ver. 2.2.2 [90]. Dot plot analysis was performed using in silico Molecular Cloning Genomics Edition ver. 6.0.11D. A heat map derived from the pairwise amino acid identity matrix tables calculated by in silico Molecular Cloning Genomics Edition ver 6.0.11D was generated using the hierarchical clustering method calculated by Cluster 3.0 [91] and visualized by Java Treeview ver 1.1.6r4 [92].

### Biofilm assays

Biofilm assays were performed as previously described [93]. Briefly, the overnight culture in BHI broth was 1,000-fold diluted with fresh BHI broth supplemented with (or without) 1% glucose. After 24 h at 37 °C incubation in a TrueLine Cell Culture 96-well plate (Nippon Genetics, Tokyo, Japan), the bacterial culture supernatant was discarded. After the wells were washed with 100 μl of Dulbecco’s PBS (−) three times, the biofilm was stained with 1% crystal violet (Nacalai Tesque) (*w*/*v*ol) at room temperature for 30 min. The dye solution was discarded, and the plate was washed with 100 μl of Dulbecco’s PBS (−) three times. The dye was extracted from biofilm with 70% ethanol containing 1% HCl. The OD_590_ was measured with a VersaMax tunable microplate reader (Molecular Devices, CA).

### Nucleotide sequence accession number

The whole genome sequences of *S. caprae* JMUB145, JMUB590 and JMUB898 were deposited in GenBank (NCBI) under the accession numbers AP018585, AP018586 and AP018587, respectively.

## Additional files

Additional file 1: **Table S1.** List of *Staphylococcus* strains used in phylogenetic analysis (XLSX 14 kb)

Additional file 2: **Table S2.** Genome information of *S. caprae* JMUB898*, S. caprae JMUB590, S. caprae JMUB145, S. epidermidis RP62a and S. capitis TW2795. (XLSX 1515 kb)*

## Abbreviations

BHI: Brain heart infusion
CoNS: Coagulase-negative staphylococci
PIA: Polysaccharide intracellular adhesin
PSM: Phenol soluble moduline
SCC*mec*: Staphylococcus Cassette Chromosome mec
SNPs: Single-nucleotide polymorphisms
TSB: Tryptic soy broth
WTA: Wall teichoic acid
